# Electroretinographic responses to periodic stimuli in primates and the relevance for visual perception and for clinical studies

**DOI:** 10.1017/S0952523824000038

**Published:** 2024-11-11

**Authors:** Jan Kremers, Cord Huchzermeyer

**Affiliations:** Section for Retinal Physiology, University Hospital Erlangen, Erlangen, Germany

**Keywords:** electroretinogram, repetitive stimuli, psychophysics, retino-geniculate pathways, chromaticity, luminance

## Abstract

Currently, electroretinograms (ERGs) are mainly recorded while using flashes as stimuli. In this review, we will argue that strong flashes are not ideal for studying visual information processing. ERG responses to periodic stimuli may be more strongly associated with the activity of post-receptoral neurons (belonging to different retino-geniculate pathways) and, therefore, be more relevant for visual perception. We will also argue that the use of periodic stimuli may be an attractive addition to clinically available retinal electrophysiological methods.

## Introduction

Imagine lightning during a thunderstorm in the night. In “Martin Chuzzlewit,” Charles Dickens describes the climax and post-climax of the lightning as follows: “… A brightness so intense that there was nothing else but light; and then the deepest and profoundest darkness…” Indeed, when the observer is dark-adapted, the flash of a nearby lightning bolt appears so bright that it is impossible to perceive its color, form, or distance. Even its direction is difficult to guess by the observer. The compression of energy within a very short time, particularly when the visual system is dark-adapted, probably prohibits the acquisition of any useful visual information. With a dilated pupil a 5 ms, 10 cd.s/m^2^ flash will probably drive retinal neurons into saturation and display response characteristics that do not exist under more natural conditions.

The electroretinogram (ERG) is a mass potential of retinal origin that can be measured non-invasively and that is elicited by visual stimuli. Currently, ERGs are most often recorded while stimulating with short (< 5 ms) flashes. Strong flashes, for example, with the mentioned 10 cd.s/m^2^ strength, are routinely used in clinical electrophysiology to diagnose and monitor retinal disorders. This convention is partially caused by the requirement to obtain reliable and reproducible results that are comparable with data obtained at other locations. The first publication of standards for full-field flash electroretinography by the International Society for Clinical Electrophysiological Society of Vision (ISCEV) was groundbreaking and has laid the foundation for the clinical application of electrophysiology in vision. The ISCEV standards for obtaining ERGs with flashed stimuli are regularly updated (Robson et al., 2022) and enable a comparison of recordings obtained from different laboratories and clinics. Although the strong flash ERG may have substantial clinical value, it is not clear that it can also contribute to better understand retinal processing of visual information that is used for visual perception.

For several years, immense progress has been achieved in the field of retinal imaging. Optical coherence tomography (OCT) and adaptive optics (AO) enable a detailed view of the retina at high resolution and in three dimensions. OCT angiography can also show retinal perfusion (Gao et al., 2016; Liu et al., 2020), and AO and OCT can demonstrate morphological changes, such as changes in the outer segment length, that are directly associated with photoreception (Pandiyan et al., 2020; Pedersen et al., 2023). Thus, imaging does no longer measure only structure, but also function. As a consequence, the number of clinical applications for visual electrophysiology is decreasing. For example, the diagnosis of occult macular dystrophy was classically diagnosed with multifocal ERG, but the changes in the outer retinal layers responsible for the decrease in mfERG amplitudes now can be directly seen on an OCT scan (Ahn et al., 2013; Nakanishi et al., 2015; Huchzermeyer et al., 2023). The ERG remains most useful when it is correlated with retinal signal processing in healthy and diseased retina.

In the days when stimuli in ERG recordings were mainly delivered by optical benches, flashes were probably relatively easy to create with mechanical shutters. But flashes may elicit transient responses with properties that are possibly not strongly related to the retinal neuronal cascade that leads to visual perception. As a result, measured ERGs can often neither be correlated with visual perception nor with psychophysical data. Indeed, until the 1970s, the number of observations, where ERG recordings and psychophysical data could be correlated, was frustratingly sparse (Armington, 1974).

The advent of monitors and LED stimulators offered the opportunity to create a larger variety of spatial and temporal stimuli. With LEDs, nearly any temporal stimulus including periodic presentations can be produced because they can be driven in the kHz range.

In the current review, we argue that ERG responses to periodic stimuli may be more strongly correlated with visual information processing in the neuronal retina and thus with visual perception than flash ERGs. This may be because they allow the neural retina to reach a steady state response. When using repetitive stimuli, the first few seconds of recordings are often discarded because they are considered as “onset artifacts.” Thus, the transients that are elicited by step changes in photoreceptor excitation, and that are explicitly recorded in flash ERGs, are regarded as artifacts when recording ERGs to repetitive stimuli.

It is the purpose of the present review to present recent developments in ERG methods that go beyond the ISCEV standard ERGs and help to improve our understanding of retinal signal processing. These developments are based on four main pillars which are often used in combination: First, the use of repetitive stimuli rather, than flashes, may show a closer relationship with visual processing in the retina than currently achieved with flashed stimuli. Second, the additional use of spectrally complex stimuli directed at specific photoreceptor types and subtypes or at post-receptoral pathways may reveal correlations with visual processing in the retino-geniculate pathway. Third, as the retino-geniculate pathway is generally believed to carry the neural signals used for visual perception, ERG responses can be correlated with psychophysical data. Finally, these results may be used to study disease-related functional changes and to improve diagnosis and/or monitoring of retinal diseases. For patients, altered ERG responses would then be particularly relevant when they directly reflect physiological processes and pathophysiological alterations in pathways that are important for visual perception. Building on existing knowledge about the correlation between ERGs and psychophysical data (sections “Considerations for comparisons of ERG and psychophysical data” and “The flash ERG and psychophysical data”), we will then elaborate on why ERGs to periodic stimuli may be more strongly correlated with psychophysical thresholds and how techniques that aim at isolating specific photoreceptor types can be used to further enhance the potential of periodic stimuli (section “The responses to periodic stimuli, the correlation with psychophysical data and clinical relevance”).

## Considerations for comparisons of ERG and psychophysical data

When comparing ERG and psychophysical data some aspects should be taken into account:

### The origins of the ERG include non-neuronal processes

**Figure 1.**
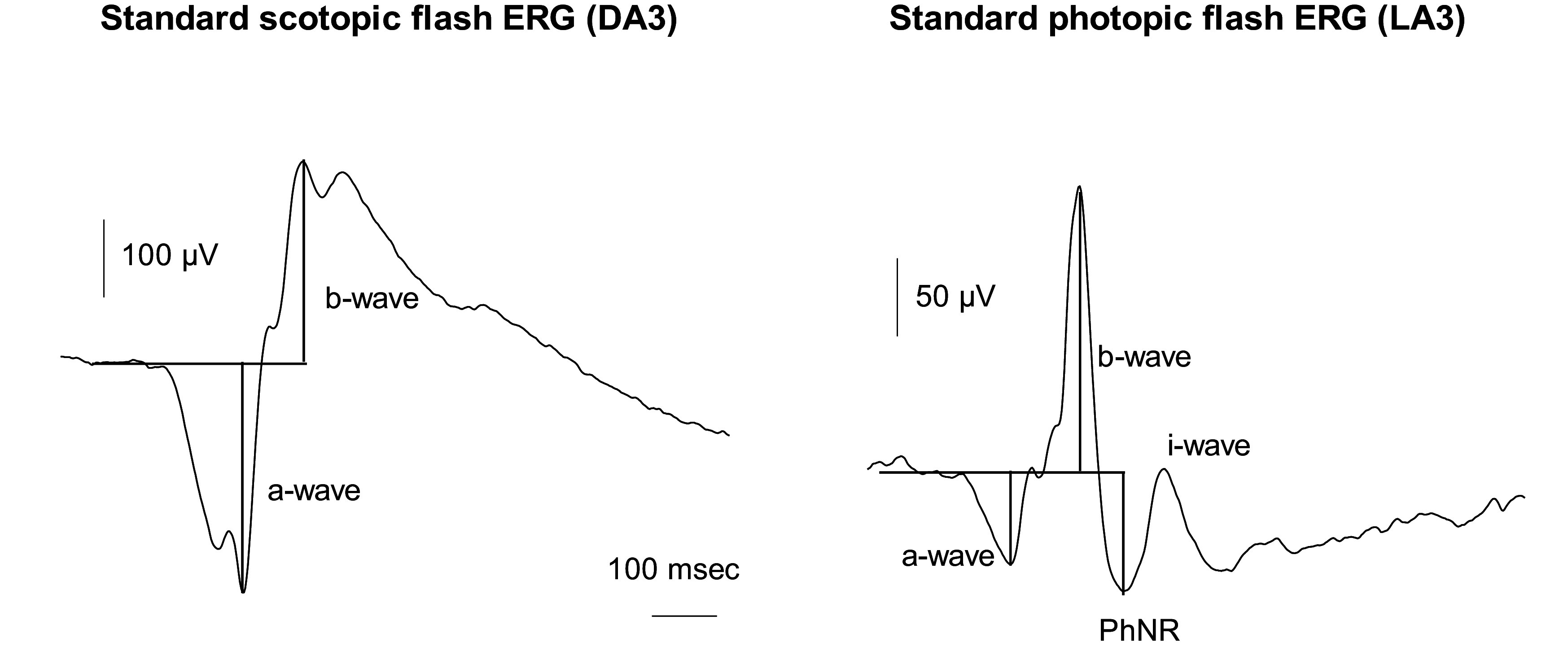
Examples of flash ERGs measured with normal healthy subject. The left trace is the response to a 3 cd.s/m^2^ flash in a dark-adapted (scotopic) state (DA3). On the right, the response to a 3 cd.s/m^2^ flash is shown during light adaptation (LA3; photopic). The different components are shown. For more details see Frishman et al. (2018) and Robson et al. (2022).

The conventional flash ERG contains several components (see Fig. 1): an early negativity (the a-wave) followed by a strong positive peak (the b-wave). On the ascending flank of the b-wave oscillatory potentials (OPs) can be observed. Under photopic conditions, the b-wave is followed by a broad negative trough that is called the photopic negative response (PhNR). The different components originate in different retinal cells (Frishman, 2006). The a-wave originates in the activity of the photoreceptors and off-bipolar cells; the b-wave reflects on-bipolar cell responses and the PhNR those of retinal ganglion cells. However, although neuronal activity is at the origin of the flash ERG they probably include secondary processes in non-neuronal cells that may influence the response properties.

The peak times of the flash ERG components (between about 20 ms for the a-wave and up to about 150 ms for the PhNR and other late components under photopic conditions) are too long to have a direct influence on perception. A simple stimulus can elicit a motoric reaction (i.e., including a decision process) after about 300 ms (Murray & Parry, 2023). Retinal Ganglion cells of the magnocellular pathway may show a maximal response to a small stimulus displacement with about 30 ms delay (Lee et al., 1993). Thus, the delay times of at least some ERG components suggest that ERGs do not directly reflect neuronal responses (although the leading edge of the a-wave is thought to originate directly and solely in photoreceptor activity). Retinal glial cells (Müller cells) are probably involved. And if glial cells are involved, the responses possibly have little relevance for visual perception. Components like the c-wave or the light peak are even much slower. They will not be considered in this review.

The origins of the ERG responses to repetitive stimuli still have to be established. Bush and Sieving (1996) found that the steady state response to 30 Hz flashes is probably homolog to the b-wave of the photopic flash ERG, suggesting a delay of about 40 ms. In conclusion, ERG responses may involve non-neuronal activity, which should be considered when comparing ERG responses with visual responses in the visual system and with psychophysical data.

### ERG measurements are often performed using psychophysically supra-threshold stimuli

A comparison of ERG responses with psychophysical data can be generally performed in two ways (Armington, 1977): First, the ERG amplitudes can be directly compared with psychophysical sensitivities. Second, an ERG response threshold can be defined and the stimulus conditions for reaching this threshold can be compared with psychophysical thresholds. The latter procedure is generally more reliable but also more time-consuming. In both cases, it should be considered that the stimuli during the ERG measurements may be psychophysically supra-threshold. (On the other hand, reliable ERGs can be measured using sinusoidal stimuli that are beyond the psychophysical flicker fusion frequency (see section “Photoreceptor isolating stimuli (silent substitution)”) indicating that subthreshold stimuli sometimes can elicit ERG responses.) Ideally, the stimuli employed in the ERG measurements have contrasts that are close to or encompass the psychophysical thresholds. However, the signal-to-noise ratio (SNR) in the ERGs may be too low to obtain reliable data when using stimuli that are close to the psychophysical threshold. It then may be important to consider the relationship between ERG amplitude and stimulus strength. If this relationship is linear both can be directly compared by using the slopes of the linear regressions. When using sinusoidal modulation, we found a linear relationship for a large number of stimuli (Usui et al., 1998; Kremers & Scholl, 2001). However, if the relationship is not linear (e.g., because of saturation) then this may affect the comparison with psychophysical data. A possible strategy would then be to search for a satisfactory mathematical description of the amplitude data as a function of stimulus strength. A Michaelis–Menten function, of which the Naka-Rushton function is a special case, has been found to describe response amplitudes as a function of stimulus strength for a large range of adaptation conditions (Valeton & van Norren, 1983). The ERG amplitude threshold should be chosen so that it is reached with stimuli that are close to those for the psychophysical threshold.

## The flash ERG and psychophysical data

In his book “The Electroretinogram” Armington (1974) included a chapter on the relationship between psychophysical and ERG data. There he wrote: “The electroretinogram is unique because its components allow the experimenter to follow several separate retinal activities, while recording is performed with a minimum of discomfort to the subject. Furthermore, the subject may make verbal reports or judgments regarding the same stimulus, which was used to elicit the electroretinogram. It is thus possible to relate the visual appearance of a stimulus to the underlying physiological processes. The full potential for doing this in a sophisticated manner, however, has not been yet realized.”

We propose that flashes are often too strong to elicit visually relevant retinal responses. As mentioned above, a 10 cd.s/m^2^ may result in a retinal illuminance of about 100,000 td. With shorter flashes, the retinal illuminance would increase proportionally to maintain the same flash strength. They are often extremely unpleasant for the observer and they elicit strong blink reflexes that elicit responses that interfere with the ERG. Such stimuli are probably beyond the natural modus operandi of the retina for transmitting useful visual information. Responses of many retinal ganglion cells, particularly of those belonging to the magnocellular pathway, display strong saturation (Kaplan & Shapley, 1986; Lee et al., 1990a) so that, at least at the level of the retinal output, a relationship between stimulus properties and neuronal response may not be straightforward. In addition, the neuronal responses to very strong stimuli often show bursts of action potentials that are possibly influenced by the neurons’ refractive period (Lankheet et al., 1989) indicating that they are influenced by extremely pronounced nonlinearities that may not be present at psychophysical threshold. There are indications that the bursty response is related to oscillatory potentials (OPs) in the rat retina (Haq et al., 2023), indicating that the OPs are a sign of response overload.

**Figure 2.**
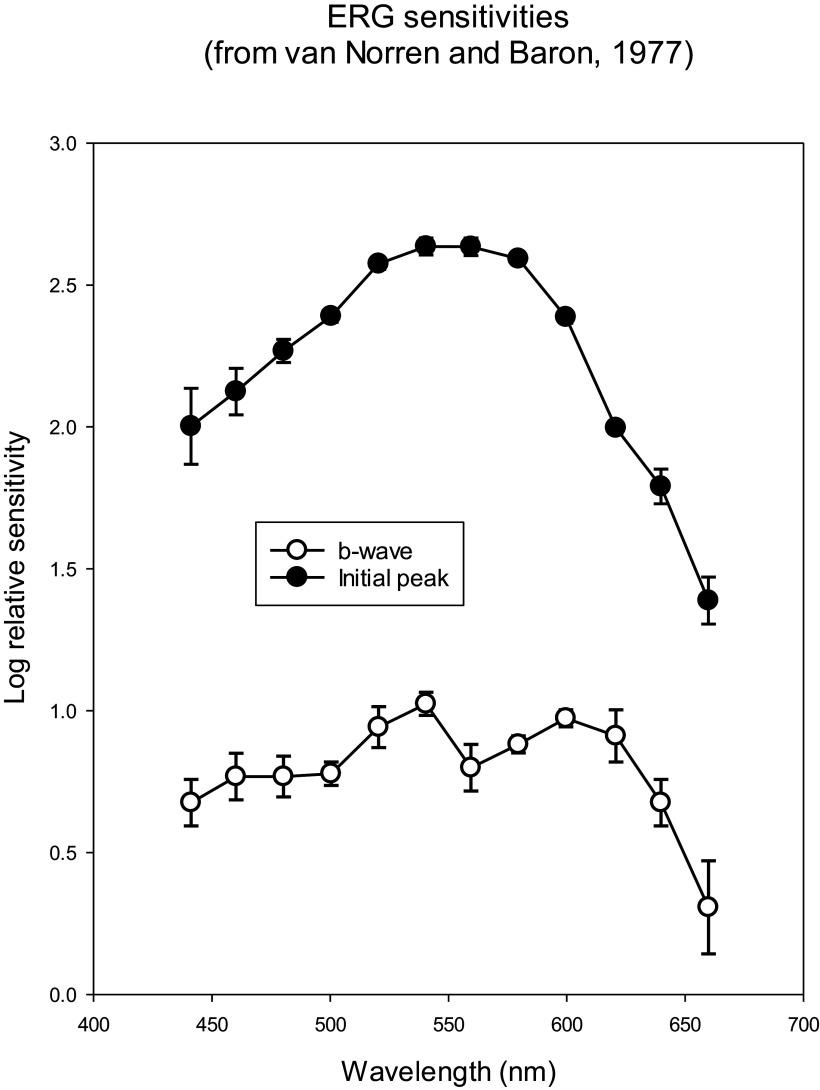
Results from local intraretinal ERG recordings at different wavelengths in cynomolgus monkeys. The plots show sensitivities (defined as the inverse of the stimulus strength for a threshold response amplitude) as a function of stimulus wavelength. The upper plot (closed symbols) shows the sensitivities of an initial peak in the response. This curve shows similarities with the *V_λ_.* The lower plot (open symbols) shows the sensitivities of the b-wave. The b-wave sensitivity displays notches at about 500 and 560 nm, suggesting that the b-wave sensitivity is at least partially determined by cone opponent processes. The two plots were shifted along the vertical axis for clarity. Data are redrawn from van Norren and Baron (1977).

**Figure 3.**
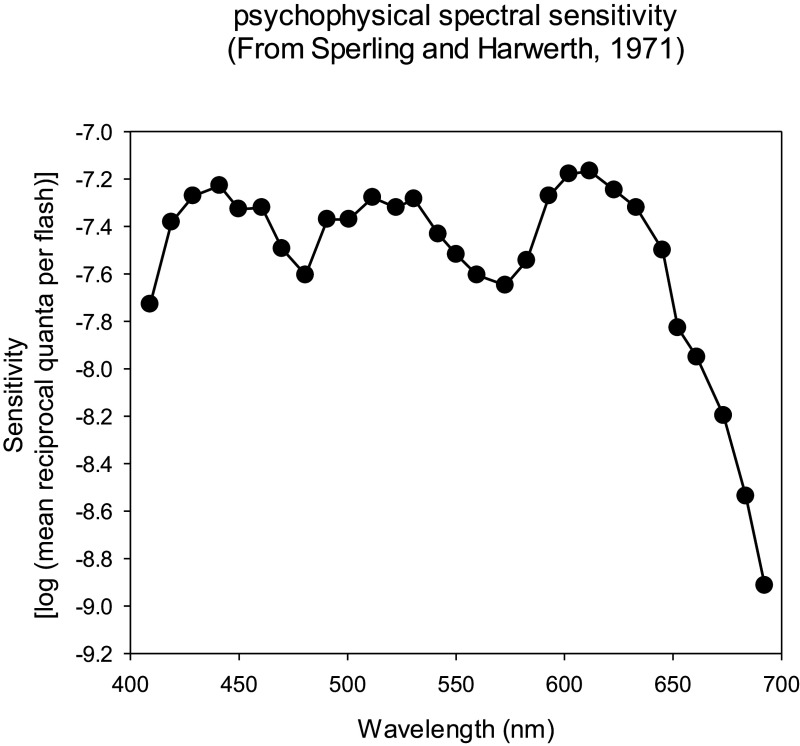
Mean psychophysically measured spectral sensitivity for incremental flashes in a rhesus monkey. Observe the same three peaks and the notches at about 490 and 560 nm similar as in the ERG amplitudes shown in the lower graph of Fig. 2. Data redrawn from Sperling and Harwerth (1971).

The transient nature of the flash ERG is another factor that may make a comparison with psychophysical data difficult. Many psychophysical procedures involve continuous stimulation so that a comparison with transient ERG responses may not be adequate. However, there may be exceptions, particularly when light-adapted conditions are used (i.e., upon a photopic background). One example of such an exception may be the spectral sensitivities of ERGs and psychophysical increment thresholds for flashes upon a background. The b-wave of the flash ERG measured in monkeys can show a notch at about 580 nm (van Norren & Baron, 1977; Mills & Sperling, 1990) indicating the involvement of cone opponent processes (Fig. 2). These spectral sensitivities were obtained with long flash durations (van Norren and Baron: 400 ms; Mills and Sperling: 70 ms), where the d-wave is not superimposed on the b-wave (Sustar et al., 2006). Interestingly, spectral sensitivities of psychophysically measured increment thresholds were found to include cone opponent process (see Fig. 3 for psychophysical data obtained in monkeys from Sperling & Harwerth, 1971; for similar data in humans see King-Smith & Carden, 1976). This seems to be generally the case for flashes that are spatially large and of long duration; for small and short-duration flashes, the spectral sensitivity resembles the spectral luminosity function *V_λ_* (Lennie et al., 1993).

The PhNR is a late component in the photopic flash ERG and is thought to reflect ganglion cell activity (Viswanathan et al., 1999). It has been claimed that the red flashes on a blue background elicit larger PhNRs than white flashes on white backgrounds (Rangaswamy et al., 2007). The luminances of the flashes were photopically matched whereas those of the backgrounds were scotopically matched. As a result, the blue background was less luminant than the white background when expressed in photopic units. In a systematic study on ERGs that were elicited by combinations of flashes and backgrounds with different peak wavelength, the PhNR was of similar amplitude and peak time if the luminances of the flashes and the backgrounds were photopically matched (Kremers et al., 2012). This indicates that the PhNR mainly has a spectral sensitivity that matches the photopic *V_λ_.* One exception was the responses elicited by 458 nm flashes on 591 nm backgrounds (peak wavelengths) that were quite different probably because of the intrusion of rod-driven responses.

In conclusion, some components of the light-adapted flash ERG (notably the PhNR) may have *V_λ_*-like spectral sensitivities indicating that the processing resembles the cone additive interactions of the magnocellularly based luminance pathway in the retina. The b-wave amplitudes to relative long flashes show indications of cone opponent processing. The correlations are, however, not very strong and they are indirect.

Please note that the correlation between the amplitudes of the flash ERG components and the *V_λ_* spectral luminosity function is only present in light-adapted ERGs, that is with backgrounds that are in the photopic range. In these conditions, the Weber contrasts (or Weber fractions) of the flashes relative to the background are possibly in a physiological range. Strong flashes with large Weber fractions (in dark-adapted state they are infinite) may evoke large ERG signals with good SNRs, but they may not be suited for relating the responses to post-receptoral processes that are relevant for vision.

## The responses to periodic stimuli, the correlation with psychophysical data and clinical relevance

Periodic stimuli can nowadays be generated relatively easily for instance with LED stimulators because the outputs of the LEDs can be controlled with high luminance resolution and updated in KHz ranges. Therefore, the waveforms can be determined with relatively high temporal precision up to the flicker fusion frequency of ERGs (at more than 100 Hz for certain conditions (Aher et al., 2019)) and of psychophysical detection (up to about 100 Hz; see Fernandez-Alonso et al., 2023). A large number of LEDs with different emission spectra (i.e., colors), spanning the complete visual spectrum between ultraviolet and infrared, are available. With organic light-emitting diodes (OLEDs), the emission spectrum is can be chosen. As a result, modulation of luminance and chromaticity, temporal frequency, and stimulus waveform can be controlled with great precision.

Repetitive stimuli may elicit steady-state ERGs that often contain only a limited number of harmonics in the frequency domain after Fourier transform. The steady-state response may involve other retinal mechanisms and pathways than those responses elicited by flashes or pulses with relatively long inter-stimulus time intervals. If the stimulus favors the response in a single post-receptoral retino-geniculate (magno-, parvo, or koniocellular) pathway, then the signature of these responses may also be detected in the ERG.

In this section, several types of repetitive full-field stimuli and the properties of the ERGs, that are elicited by these stimuli, will be discussed. These properties will be compared with psychophysical data that are mediated by different retino-geniculate pathways. Furthermore, some preliminary data for clinical applications will be provided.

### ERG responses to sinusoidal stimuli

Sine wave stimuli were used in several studies. The stimuli can be luminance or chromatic modulation or combinations of the two.

#### Luminance stimuli at different temporal frequencies

**Figure 4.**
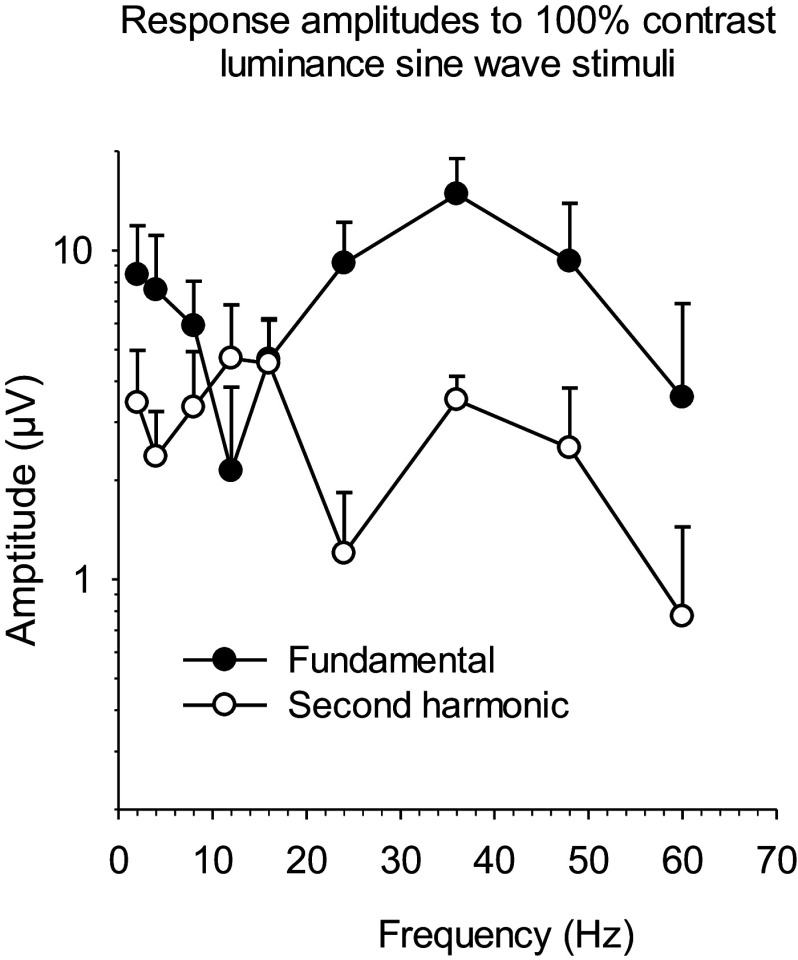
Amplitudes of the fundamental (closed circles) and second harmonic (open circles) components of observers’ responses to luminance sine-wave stimuli with 100% Michelson contrast. The fundamental component shows a minimum of about 12 Hz. The second harmonic displays a maximum at this frequency. Replotted from Pangeni et al. (2010).

The ERG responses to sinusoidal luminance modulation were measured in several studies in human subjects (Burns et al., 1992; Odom et al., 1992; Pangeni et al., 2010; McAnany & Park, 2019) and in non-human primates (Kondo & Sieving, 2001; Viswanathan et al., 2002). In all studies, a conspicuous amplitude minimum of the first harmonic (fundamental) response component was observed at about 12 Hz (see Fig. 4). The second harmonic component displayed a maximum at this frequency and even exceeded the first harmonic indicating that the ERG displayed a frequency doubling at that frequency (Pangeni et al., 2010). Kondo and Sieving (2001) proposed that the minimum was caused by the cancelation of Off- and On-responses (they were able to study the two responses separately by blocking them selectively; APB blocked the synaptic transmission of photoreceptors to On-bipolar cells whereas PDA blocked the transmission to Off-bipolar cells). Pangeni et al. (2010) proposed that the response to sine waves would be determined by two independent components with different response waveforms, one dominating at low temporal frequencies and the other at high frequencies. At about 12 Hz, the two components were of similar amplitude, and the simultaneous appearance of the two components led to the frequency doubling.

Interestingly, patients with x-linked Duchenne Muscular Dystrophy (DMD), which also affects the retina, were found to have an asymmetric alteration of On- and Off-responses (Barboni et al., 2013) as measured with sawtooth stimuli (see also section “Responses to sawtooth and square wave stimuli”). Furthermore, they were found to lack the amplitude dip at 12 Hz when luminance sine-waves were displayed (Barboni & Kremers, unpublished data). This finding is in agreement with the hypothesis of Kondo and Sieving because an On–Off asymmetry would prevent the two signals to cancel each other out. Kondo and Sieving’s proposal and ours are not mutually exclusive. Possibly, the On- and Off-signals contribute to one of the two independent components as defined by Pangeni et al. The On–Off asymmetry would then result in an amplitude increase of this particular component. The results would again be that the minimum at 12 Hz is less conspicuous or absent. It would be interesting, to repeat the measurements, performed in DMD patients, in other patient groups that are known to have On–Off asymmetries, such as X-linked retinoschisis (XLRS; see also section “Responses to sawtooth and square wave stimuli”) or congenital stationary night blindness (CSNB). The expected combination of On–Off asymmetry and the absence of the 12 Hz dip has been confirmed for one CSNB patient (McAnany et al., 2013). Interestingly, in these diseases, the synapse between photoreceptors and bipolar cells is involved.

The responses to luminance sine-wave stimuli show an additional interesting non-linearity: for stimuli in a narrow range between about 33 and 38 Hz, they may show period doubling (or frequency halving) so that the responses to odd and even stimulus periods show different amplitudes (Alexander et al., 2005, 2008; Alexander & Raghuram, 2007; McAnany et al., 2019). Period doubling has been attributed to nonlinear feedback mechanisms at the synapse between photoreceptors and bipolar cells (Crevier & Meister, 1998). This stimulus may also have clinical application because alterations in period doubling frequencies were found to be smaller in diabetes patients with no diabetic retinopathy or with mild non-proliferative diabetic retinopathy (McAnany et al., 2019).

#### Combined luminance and chromatic stimuli

Many stimuli contain both luminance and chromatic modulation. In practice, two types are often used: heterochromatic modulation, where the chromatic and luminance content in the stimulus can be quantified, and photoreceptor-specific stimuli using the silent substitution paradigm.

##### Heterochromatic modulation

**Figure 5.**
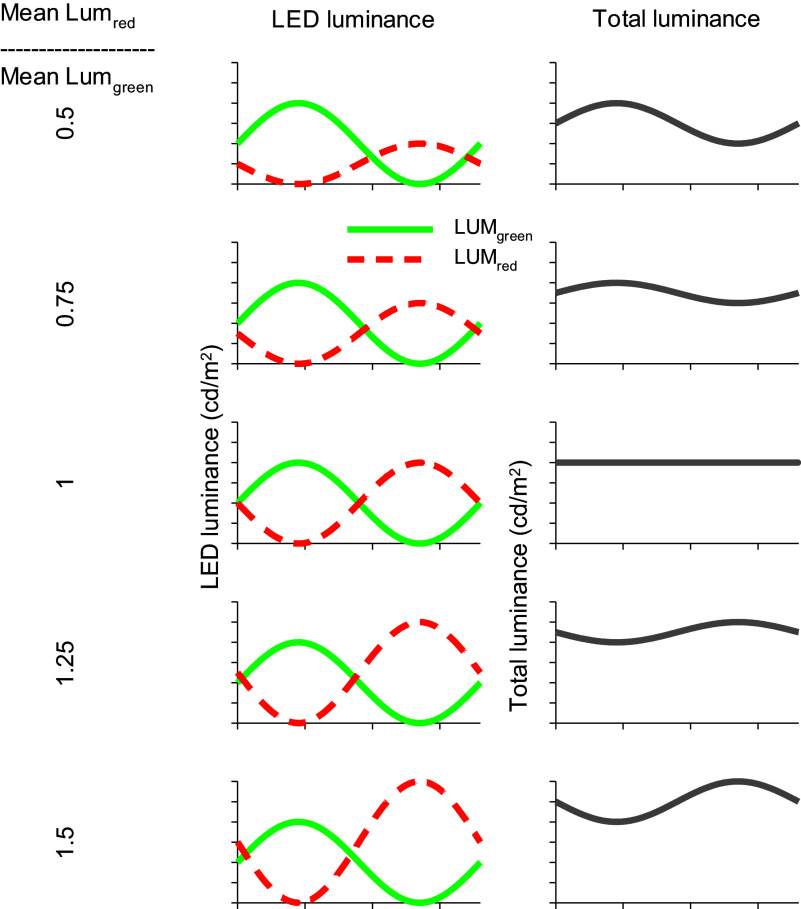
A description of the heterochromatic flicker photometry paradigm. Two differently colored stimuli are modulated in counterphase at equal contrast (mostly 100% as depicted in the figure). The mean luminance of the reference stimulus (green in this case) is kept constant whereas the mean luminance of the test stimulus (red in the present case) is varied. The luminance modulation is zero when the two stimuli have equal mean luminances.

During heterochromatic modulation, the outputs of two differently colored light sources are modulated in counterphase. Heterochromatic stimulation is used to record ERGs as an electrophysiological pendant of the psychophysical heterochromatic flicker photometry (HFP) or heterochromatic modulation photometry (HMP) procedures. In HFP, the two colors are modulated at high temporal frequency (generally 20 Hz and higher) and often with maximal (100% Michelson) contrast. The mean luminance of one color (the test) is changed whereas the other (reference) color remains constant. When the percept of flicker is minimal (Kaiser, 1988) then, by definition, the two colors are isoluminant (Fig. 5). If different monochromatic test colors are used with constant reference colors (e.g., white light) then the spectral sensitivity can be obtained, which, for a standard observer, is the *V_λ_* (spectral luminosity function). The spectral sensitivities obtained from different healthy trichromatic observers can show a considerable variability.

In ERG measurements a minimum in the fundamental ERG component is found to coincide with individual perceptual minimal flicker. Thus, the electroretinographically and psychophysically obtained spectral sensitivities are generally identical. This procedure has been used extensively by Gerald Jacobs and colleagues (Jacobs et al., 1996b). They also found that the abovementioned individual differences in the psychophysical HFP procedure are reflected in the ERGs (Neitz & Jacobs, 1986, 1990; Jacobs & Neitz, 1993a, 1993b). They further used this procedure to determine the spectral luminosity function in various mammal species (Jacobs & Neitz, 1984; Neitz & Jacobs, 1984; Jacobs et al., 1991, 1993; Jacobs & Deegan II, 1993; 1997; Deegan II & Jacobs, 1996; Jacobs et al., 1996a). The HFP procedure has the advantage that: (1) thresholds are determined rather than amplitudes (see above) and (2) the fundamental component can be obtained with high precision.

Another procedure that is similar to but not identical to the HFP procedure is heterochromatic modulation photometry (HMP) where, differently from HFP, the mean luminance of the two colors is constant and the modulation depth is changed and a threshold is obtained (Pokorny et al., 1989). The thresholds for different ratios of the modulation (Michelson) contrast in the two colors can then be obtained. Alternatively, the ratios can be varied and the ratio range, where no flicker is perceived is determined. As mentioned, in the HMP procedures, the mean luminances are constant and thus the mean state of adaptation remains unaltered. This is an advantage relative to the HFP procedure where the mean state of adaptation is varied. Thus, a confounding with the state of adaptation is absent in the HMP procedure and the state of adaptation can be used as an independent invariant the influence of which can be studied.

**Figure 6.**
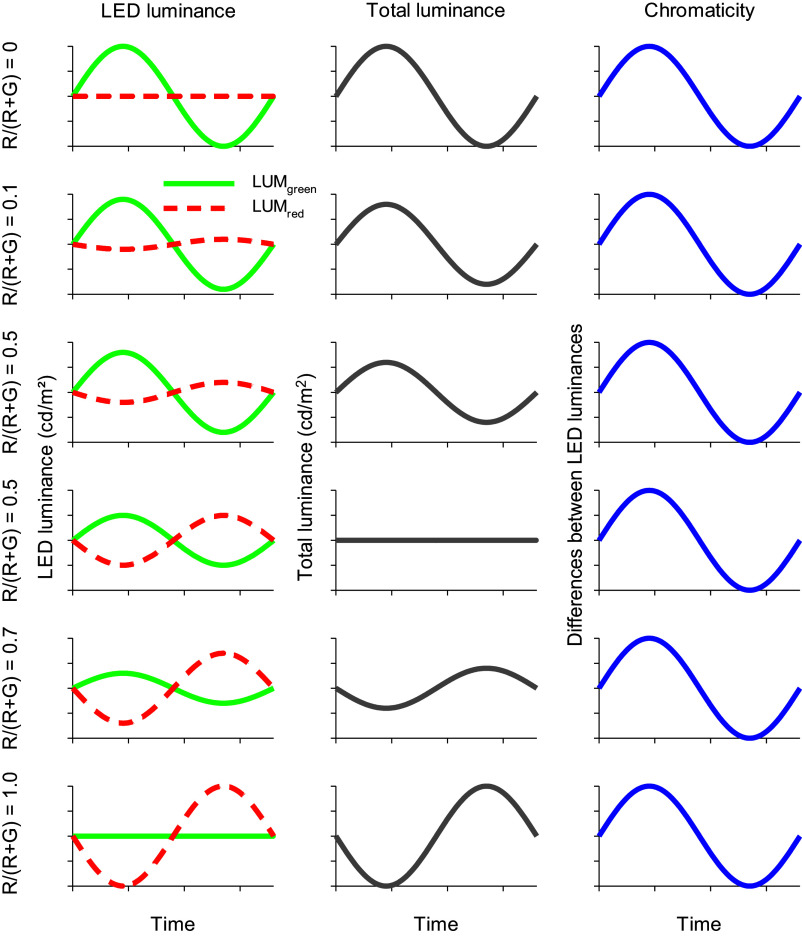
Description of the HMP procedure. Left column: Luminance of the red (Lum_red_) and green (Lum_green_) LEDs (arbitrary values in cd/m^2^) for six different conditions in which the fraction (*F_R_*) is varied. *F_R_* is defined as the fraction of red contrast (*R*) over total contrast (*R + G*): *F_R_ = R/(R + G).* In our experiments, the total contrast was 100%. Middle column: Luminance modulation elicited by the stimuli (defined as Lum_green_ + Lum_red_). The luminance modulation depth (and thus luminance contrast) depends on stimulus conditions. Luminance contrast is 0% when *F_R_ = 0.5.* At this minimum the phase of the luminance modulation shifts by 180°; when *F_R_ < 0.5* the luminance follows the output of the green LED; when *F_R_ > 0.5* the luminance follows the output of the red LED. Right column: Chromatic modulation (defined as Lum_green_-Lum_red_) for the different conditions. Neither contrast nor phase of the chromatic modulation changes with *F_R_.*

In a series of ERG measurements, we employed HMP stimuli with red and green stimuli. We varied the contrast in the two lights (*R* is red contrast and *G* is green contrast), according to the HMP procedure, while keeping the total modulation (*R + G*) constant at 100%. The response amplitudes were measured for different fractions of red contrast (*F_R_ = R/(R + G) = R/100%*). Thus, when *F_R_ = 0* then only green is modulated (with *G = 100%*) and red is kept constant at the mean luminance (i.e., *R = 0%*). Only red is modulated with 100% and green is constant at its mean luminance when *F_R_ = 1.* Fig. 6 shows how luminance and chromaticity are modulated. The luminance modulation depends on *F_R_* and equals 0 when *F_R_ = 0.5*, where the modulation phase shifts by 180 degrees. This is the case for the standard observer with *V_λ_* spectral sensitivity. The isoluminance point varies between different observers, due to several factors such as individual differences in the ratio of L- and M-cone numbers, genetic variability that influence the cone absorption spectra and variability in pre-retinal absorption.

In contrast to the luminance modulation in the stimulus, the modulation of chromaticity is equal for all values of *F_R_.* Both amplitudes and phases of the chromatic modulation do not alter with *F_R_* (Fig. 6; right column).

**Figure 7.**
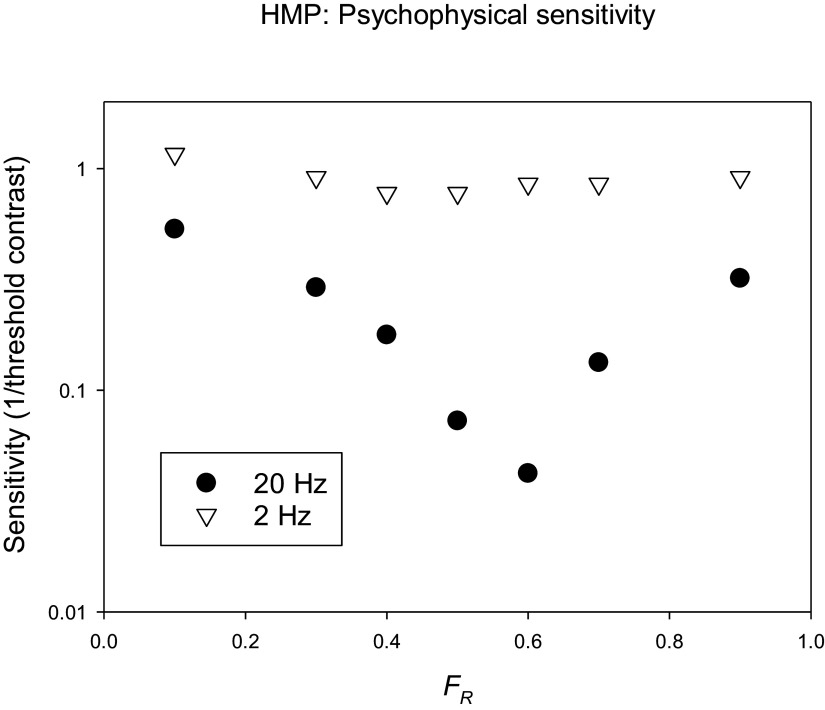
Results of psychophysical measurements with a trichromatic subject. The psychophysical sensitivity (inverse of the contrast at detection threshold) given as a function of the fraction of red contrast relative to the total contrast (*F_R_*) in the stimulus. The measurements were performed at two temporal frequencies. At 2 Hz (open inverted triangles), the sensitivities are similar for all values of *F_R_*, whereas the sensitivity shows a clear minimum at 20 Hz (closed circles). (Aher, Kremers, Huchzermeyer, unpublished data).

In psychophysical studies, at each *F_R_* the modulation contrast in the two LEDs is changed proportionally (i.e., without changing *F_R_*) to assess the flicker detection threshold (the inverse of which, by definition, is the sensitivity). At high temporal frequencies, the sensitivity strongly depends on *F_R_* (see Fig. 7, closed circles, for measurements performed at 20 Hz). At the *F_R_* value at which the sensitivity is lowest, the two colors are isoluminant. As can be seen in Fig. 6, the chromatic modulation (i.e., the modulation of an L-M cone opponent system) does not change when *F_R_* is changed. The psychophysical sensitivity, measured at 2 Hz temporal frequency, was similar for all *F_R_* values and no clear minimum was found (see Fig. 7; open inverted triangles), indicating that the sensitivity was determined by a color opponent pathway. Pokorny et al. (1989) also found less obvious minima with decreasing temporal frequencies (down to 8 Hz). It can be expected that for even lower frequencies, the minimum would be even less obvious.

**Figure 8.**
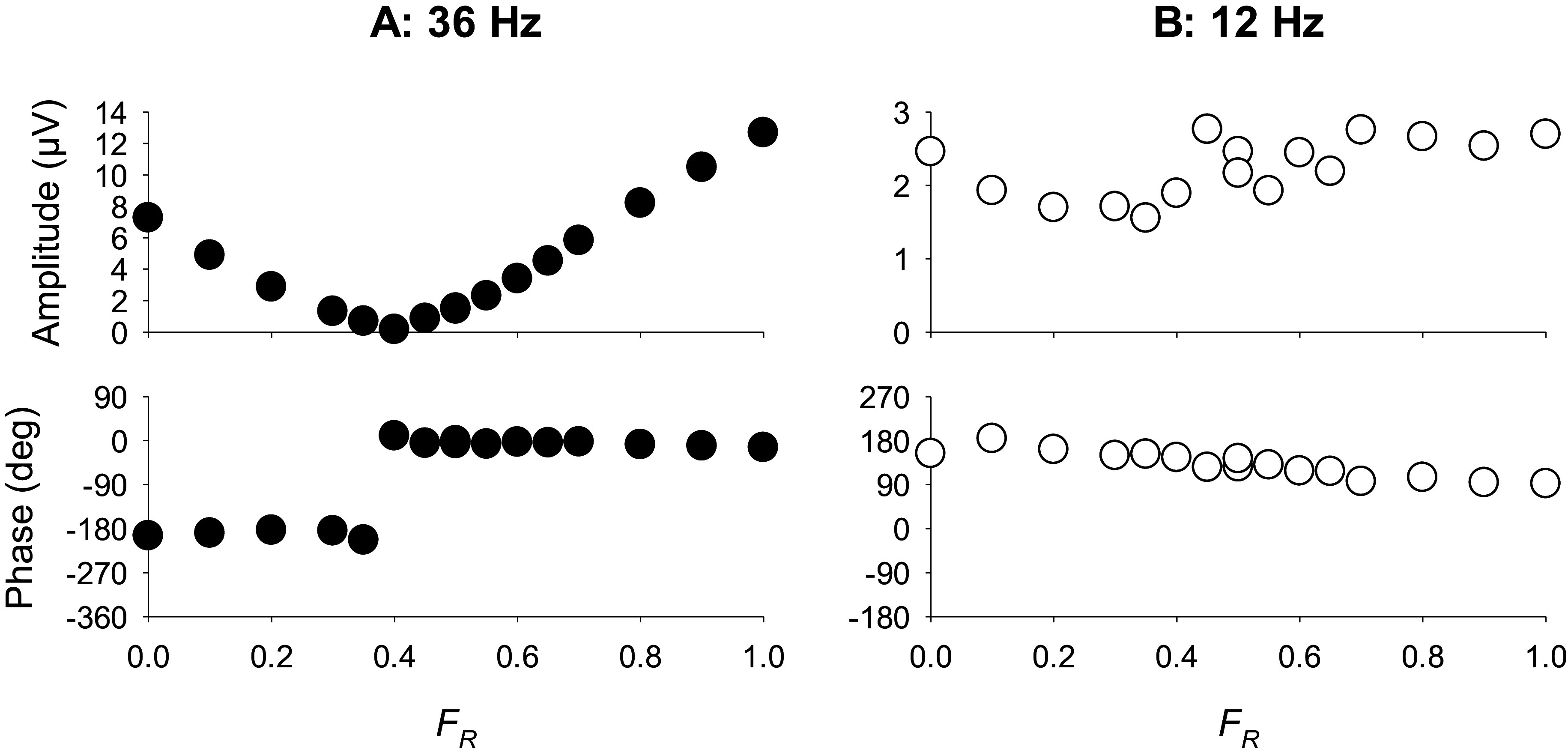
Amplitudes (upper plots) and phases (lower plots) of the fundamental components in the ERG responses measured in a trichromatic subject to HMP stimuli (as sketched in Fig. 6) as a function of the *F_R_.* The responses to 36 Hz stimuli (left plots) show a clear amplitude minimum (similar to the 20 Hz psychophysical sensitivities shown in Fig. 7). At the minimum, the response phases change by 180 degrees. The 12 Hz response amplitudes and phases (right plots) do not change strongly with *F_R_.* This was also observed in the 2 Hz psychophysics (see Fig. 7) and can be expected when the responses (and the psychophysical sensitivities) reflect cone opponent activity. Data from Kremers et al. (2010).

ERG studies using high frequency (36 Hz) HMP stimuli (see Fig. 8A) revealed that the fundamental component (that dominates the responses) was minimal at *F_R_* values where the psychophysical sensitivities are also close to minimal. The phase of the fundamental component changed by about 180 degrees at the minimum. These data indicate that the responses were determined by activity of the luminance channel (Kremers et al., 2010; Kremers et al., 2021b). In contrast, at temporal frequencies between about 8 and about 14 Hz the amplitudes and the phases of the fundamental components were fairly constant for all *F_R_* values (see Fig. 8B). This indicates that the responses were determined by the chromatic component in the stimulus and that they reflected activity of the L-M cone opponent retinal pathway.

The data indicate that psychophysical and ERG data correspond closely with each other and follow the luminance modulation in the stimulus at high temporal frequencies. The ERGs are determined by the chromatic content at lower temporal frequencies. However, the exact temporal frequencies at which the two data sets are congruent differ: luminance reflecting data can be found above about 20 Hz for psychophysical data and above about 30 Hz in the ERGs. Data reflecting chromatic pathway characteristics can be found between about 8 and 16 Hz in the ERGs whereas frequencies below about 4 Hz are needed in the psychophysics. These frequency differences can have several causes: for instance, at low temporal frequencies, rod-driven responses may influence the ERGs. In the psychophysics rod responses generally do not influence the sensitivities at photopic conditions. At high temporal frequencies, the difference may be caused by the fact that ERGs can be measured beyond the psychophysical flicker fusion frequencies (Aher et al., 2019). Retinal ganglion cells also respond to frequencies beyond the psychophysical fusion frequency. It was proposed that low-pass central filters abolished the high-frequency retinal responses (Lee et al., 1990b). In addition, it should be considered that ERG recordings are generally performed using larger stimuli than those used in psychophysical measurements, which may result in secondary deviations between psychophysical and ERG data. It was additionally found that ERGs reflecting luminance activity decrease in amplitude strongly with decreasing stimulus size whereas the amplitudes of those reflecting cone opponency are fairly constant for a range of stimulus sizes (Jacob et al., 2015; Kremers et al., 2021b).

The ERG recordings to HMP stimuli have also been performed in different patient groups revealing their possible clinical application. ERG data to a subset of stimuli described above obtained from glaucoma patients revealed phase differences with those from control subjects (Barboni et al., 2011). Patients with Duchenne Muscular Dystrophy (DMD) showed ERG responses at 12 Hz that reflected luminance activity. Female carriers and control subjects displayed responses that were determined by the chromatic content of the stimulus (Barboni et al., 2016). More recently, it was found that patients with X-linked juvenile retinoschisis (XLRS) showed luminance reflecting ERGs at 12 Hz, similar to the DMD patients. Again, female carriers showed ERGs that were determined by the chromaticity modulation (Zobor et al., 2023). Interestingly, XLRS (Zobor et al., 2023) and DMD (Barboni et al., 2013) patients display asymmetric changes in luminance On- and Off-responses (see also section “Responses to sawtooth and square wave stimuli”). It still has to be established if these results are connected.

**Figure 9.**
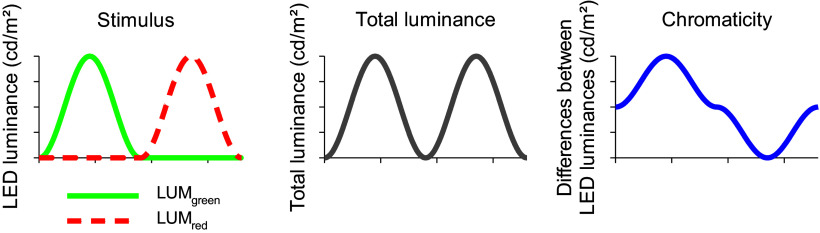
Sketch of a heterochromatic stimulus where the luminances of the red and green LEDs are alternately modulated according to raised cosine functions. The luminance modulates at approximately twice the temporal frequency of the chromatic modulation.

Another heterochromatic stimulation that uses stimuli that are not perfectly sinusoidal seems similar to the HFP and HMP methods at a first glance but stimulates the luminance and cone opponent pathways in a substantially different manner. Here, two differently colored lights are not modulated in counterphase but they are modulated alternatively according to raised cosine functions. Thus, when the red stimulus is modulating the green stimulus is off and vice versa. The consequence is that the luminance in the stimulus is modulated at twice the temporal frequency as the chromatic frequency (see Fig. 9).

**Figure 10.**
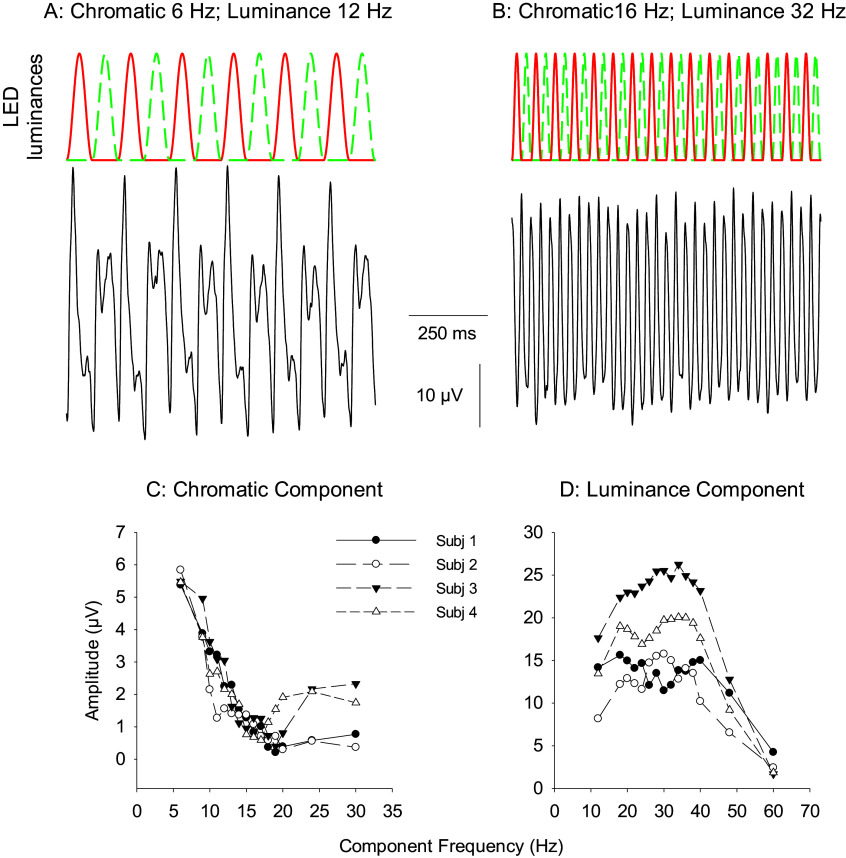
(A) Sketch of an alternating red-green stimulus with 6 Hz chromatic and 12 Hz luminance frequency. The original responses from a trichromat (subject 2) are shown in the lower part of (A). Observe that the response to the red and green LEDs differ showing that there is a substantial response to the chromaticity of the stimulus. (B) The stimulus plus responses in subject 2 for a 16 Hz chromatic; 32 Hz luminance stimulus. The responses to the red and green LEDs are very similar, indicating that the response is mainly determined by the luminance modulation. (C) Amplitudes of the response component at the chromatic stimulus frequency as a function of the component’s frequency for four different trichromatic subjects. (D) Response amplitudes of the component at the luminance frequency of the stimulus. Data redrawn from Parry et al. (2012).

This stimulus was first used in studying the responses of magnocellular (MC) and parvocellular (PC) primate retinal ganglion cells (RGCs). In the RGC recordings, it was found that the MC-cells were basically responding to the luminance component in the stimulus whereas the PC-cells were mainly responding to the chromatic component (Lee et al., 2011). This stimulus was also used in ERG recordings (Parry et al., 2012). It was found that the ERG responses displayed strong responses to the chromatic component up to a frequency of about 15 Hz. The response to the luminance component was particularly large when its frequency was larger than 20 Hz (i.e., where the chromatic component is modulated with 10 Hz) and peaked at frequencies between 30 and 40 Hz (where the chromatic modulation component is between 15 and 20 Hz). Similar to the data with HFP and HMP paradigms the results show that the ERGs reflect luminance (MC-) activity at high temporal frequencies and cone opponent (PC-) activity at intermediate temporal frequencies (see Fig. 10).

The three stimulus methods were validated by performing recordings in dichromats (protanopes and deuteranopes). Invariably, the ERG responses to the chromaticity component in the stimulus were absent in these recordings. Furthermore, the responses to the luminance components displayed the characteristics from only the present cone type, whereas the responses obtained from trichromats could be explained by agonistic interactions of L- and M-cone inputs. The responses in trichromats were generally dominated by the L-cone input which is in agreement with structural data where it was found that the human retina contained more L- than M-cones (Brainard et al., 2000).

In conclusion, ERGs elicited by heterochromatic stimuli reflect luminance activity (based on the magnocellular retino-geniculate pathway) at frequencies above about 30 Hz and (parvocellularly based) L-M opponency at frequencies between 8 and 12 Hz. At frequencies between 12 and 30 Hz, the responses to the HMP paradigm are a mixture of the two and the strength of the signals of the pathways is frequency-dependent (Kremers et al., 2021b).

The two ERG signals also have different spatial properties: The luminance reflecting ERG signal increases with increasing stimulus size. In addition, the response decreases with increasing retinal eccentricity of the stimulus. In contrast, the cone opponent signal is fairly constant for a large range of stimulus sizes and positions. A positive correlation between stimulus size and response amplitude was found when the stimuli were smaller than about 10° in diameter (Martins et al., 2016; Vidal et al., 2021; Kremers et al., 2021b). Thus, if residual retinal responses are restricted to small areas (e.g., after substantial degeneration of the retina) they possibly can be studied with stimuli that reflect activity of the L-M cone opponent channel.

It should be further mentioned that the results described in this section were obtained in human subjects while the data on neuronal responses were mainly obtained in old-world non-human primates. We found, that the ERG responses in monkeys were similar to those found in humans and the same pathway-related responses were found in the monkey ERGs (Kremers et al., 2021a). Only a detailed comparison also showed differences. Recently, a detailed connectomic electron microscopy study of retinal pathways in macaques, marmosets, and humans revealed some basic differences mainly concerning pathways that process S-cone signals (Kim et al., 2023).

##### Photoreceptor isolating stimuli (silent substitution)

With flashed stimuli, the isolation of the response of one photoreceptor system is generally obtained by using background colors and luminances that desensitize those systems that are not of interest. The flash has a wavelength content to which the photoreceptor system of interest is particularly sensitive. Thus, the response to the flash is mainly determined by this photoreceptor system. However, the isolation is not perfect because the desensitization will often not completely abolish the responsivity of the other photoreceptor systems. In addition, the flash is likely to excite one or more desensitized photoreceptors and possibly this change in excitation will lead to an ERG response. Indeed, the ISCEV extended protocol for an S-cone ERG (Perlman et al., 2020) clearly shows that the response is contaminated by responses driven by the L- and/or M-cones. In addition, although the desensitizing approach can be used (e.g., in a clinical setting) to compare the responses obtained in two subject groups (e.g., in normal subjects and patients), the responses obtained with conditions directed at different photoreceptor types cannot be compared with each other because strongly different chromatic backgrounds are used that bring the retina generally in different states of adaptation and thus in different response modes.

The silent substitution method is a method that considers the sensitivities of each photoreceptor type (more correctly each photopigment, because in some species cones can express more than one photopigments (Lyubarsky et al., 1999); in addition, photoreceptors may receive feedback signals from other photoreceptors with different photopigments (Kamar et al., 2019)) to each light source. The modulation in the different light sources is chosen such that only one photoreceptor type is modulated whereas the stimulus does not evoke a change in excitation in the other photoreceptor systems (silent substitution). The method was first described by Donner and Rushton (1959) and further developed by Estévez and Spekreijse (1974, 1982). A detailed description of the silent substitution method can be found elsewhere (Kremers, 2003).

The maximal number of photopigments that can be independently modulated equals the number of stimuli with independent emission spectra are available (independent meaning that the emission spectra of one light sources cannot be generated by a linear combination of the outputs of the others) because then the transformation from LED output to photopigment excitation is a linear matrix calculation (Martin et al., 2023; Nugent et al., 2023). For most human subjects, the number of photosensitive cells equals five (three cone types, the rods, and the ipRGCs). Therefore, stimulators must have at least five primaries when all photoreceptor types are to be stimulated independently. When the number of independent light sources exceeds the number of photoreceptors, metameric stimuli are possible where different stimuli result in identical photoreceptor responses.

The excitation in the photopigments is calculated by the multiplication of their emission spectra with the fundamentals (i.e., the pigments’ absorption spectra at the pupil thereby considering factors such as pre-retinal absorption). In order to achieve a large space of possible combinations of photopigment modulations (large gamut), the emission spectra of the light sources have to be relatively narrow band (as for instance with LEDs) and their distribution within the visual spectrum has to be well chosen (for instance by avoiding strong overlaps). Generally, the maximal photopigment excitation modulation decreases when more photopigments need to be considered because of an increasing overlap of their fundamentals.

**Figure 11.**
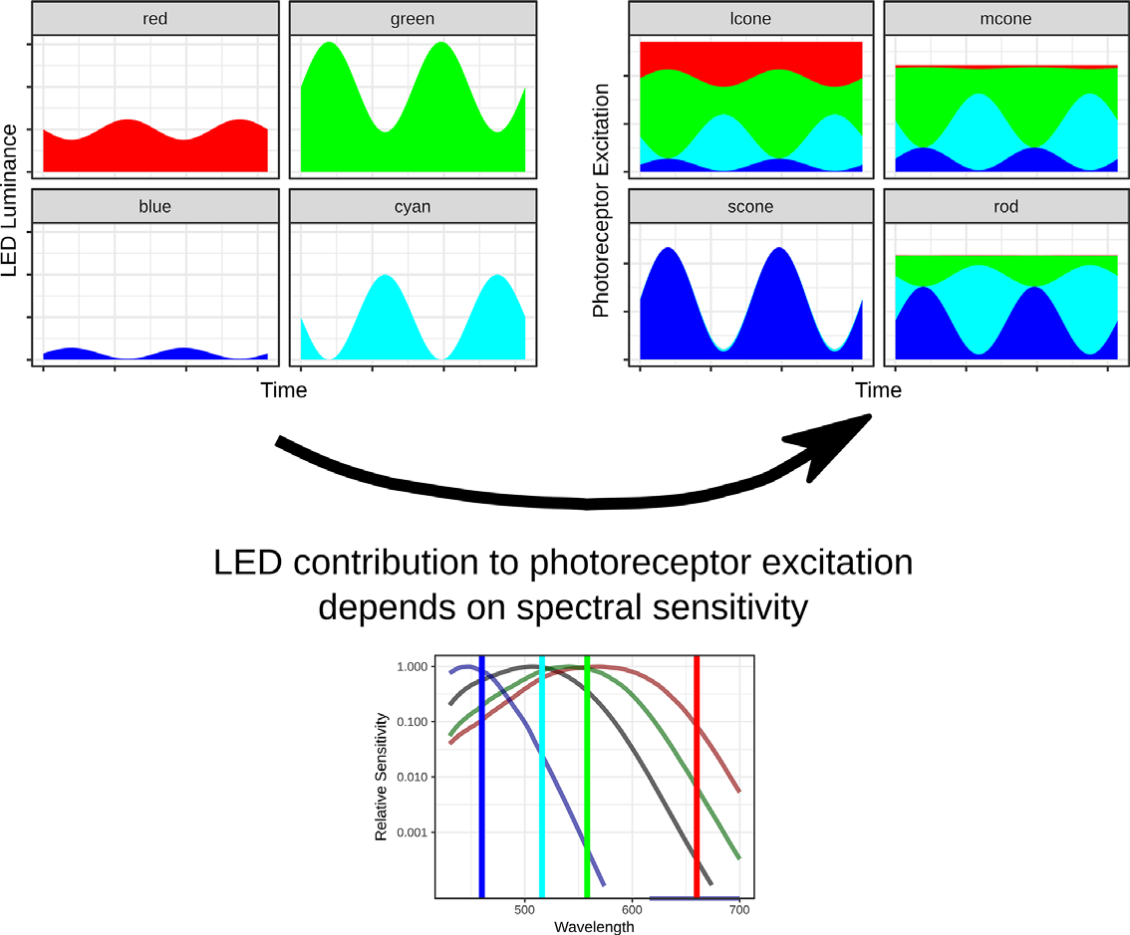
Sketch of the silent substitution method considering four primaries and four photoreceptor types (rods and three cone types). From the photoreceptor fundamentals and the emission spectra of the light sources (in this case narrow band LEDs with interference filters), the sensitivity of each photoreceptor to each LED is calculated. The luminance modulation in each LED (upper left plots) is chosen such that the sum of the photoreceptor excitations elicited by the four LEDs (upper right plots) is modulated in only photoreceptor type (in this case the S-cones). The sum of the excitations in the other photoreceptor types is not modulated (i.e., resulting in a triple silent substitution).

The silent substitution stimulus is a special case of the mentioned space of possible photoreceptor contrast combinations in which the contrasts in one or more photopigments are zero. For a photopigment isolating stimulus, the contrasts in all other photoreceptors are zero (see Fig. 11). In contrast to the selective desensitization method, with the silent substitution method theoretically a perfect isolation can be obtained, but variability in fundamentals will cause deviations. If the emission spectrum of one primary overlaps strongly with the fundamental of one photopigment but not with the others, then relatively large modulation contrasts in the isolated photopigment are possible.

The silent substitution method can be easily implemented with sinusoidal and other repetitively modulating stimuli. With repetitive stimuli, the silent substitution method can have the additional advantage that the isolation of different photoreceptor responses can be obtained without changing the mean luminances of the light sources and thus of the state of (luminance and chromatic) adaptation. As a result, the responses evoked by different photoreceptor systems can be directly compared with each other and the effects of the state of adaptation can be independently assessed. In principle, silent substitution can also be used for flashes, but it should be considered that the state of adaptation may then change with flash strength, frequency, and duration.

Measurements with dichromats can be used to validate the correctness of the stimuli: L-cone isolating stimuli did not elicit ERGs in protanopes and M-cone isolating stimuli did not lead to ERG responses in deuteranopes (Kommanapalli et al., 2014). Thus, the stimuli are indeed validated on the photoreceptor level. In contrast, the validation of the heterochromatic stimuli as mentioned in the previous section, is based on differences in the cone opponent post-receptoral processes between di- and trichromats.

ERG responses with relatively large SNRs are possible. Furthermore, photoreceptor inputs to post-receptoral pathways are well described. Thus, the impact of the stimuli on these pathways can be described and compared with the ERG responses.

**Figure 12.**
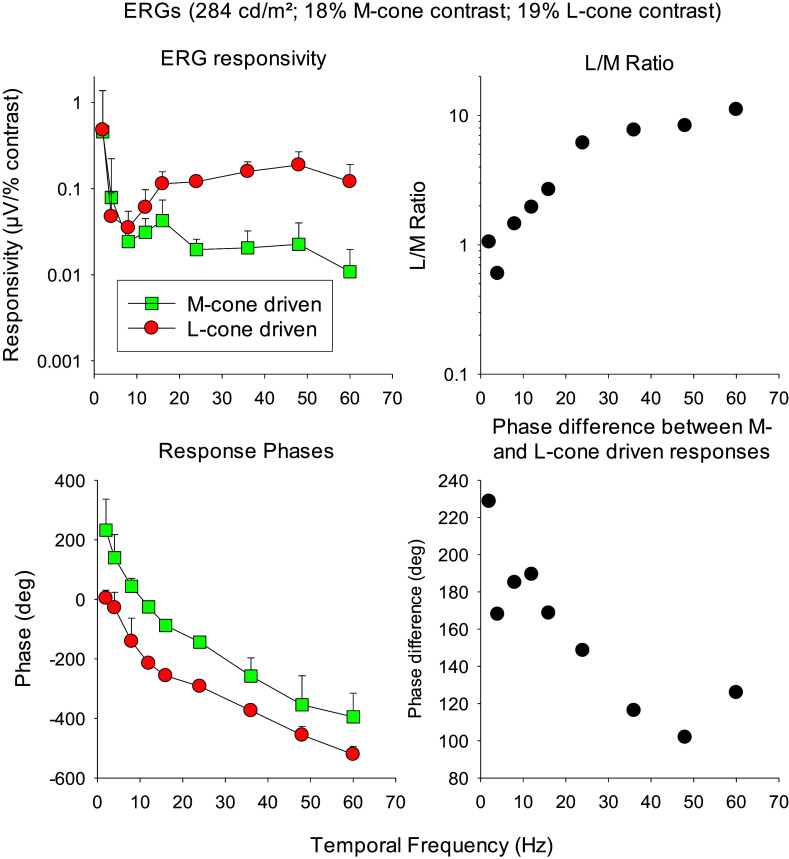
Upper left: ERG responsivity to L- and M-cone isolating sine wave stimuli versus temporal frequency. Responsivity is defined as the response amplitude divided by the used cone contrast, enabling a comparison between conditions with different contrasts. In the current case, the difference is very small (18% M-cone contrast vs. 19% L-cone contrast). Upper right: The ratio of L- and M-cone driven responsivities as a function of temporal frequency. The ratio is close to one at low frequencies and increases with temporal frequency and can be as large as 10:1. Lower left plot: The phases of L- and M-cone driven ERGs versus temporal frequency. Lower right plot: Phase difference between M- and L-cone driven responses as a function of temporal frequency. The difference is close to 180 degrees at low temporal frequencies (suggesting cone opponency) and decreases with increasing frequency. Redrawn from Kremers and Pangeni (2012).

ERG responses to L- and M-cone isolating sinusoidal stimuli show typical dependencies on temporal frequencies (see Fig. 12). The amplitudes of the first harmonic components (Fig. 12, upper left plot) show a minimum at about 10 Hz and increase at higher frequencies. They show a maximum of about 40 Hz. Although particularly the fundamental component elicited by L-cone isolating stimuli is reminiscent of the responses to luminance stimuli (cf. Fig. 4 fundamental component; this agrees with the notion that responses reflecting luminance activity are dominated by signals coming from the L-cone) the second harmonic components are generally small. At temporal frequencies above about 60 Hz the response amplitudes steadily decrease but the response can be measured up to 100–120 Hz (Aher et al., 2019). These flicker fusion frequencies are similar to those of RGCs (Lee et al., 1990b). Interestingly, the response amplitudes to L- and M-cone isolating stimuli with similar cone contrasts are of similar magnitude at low temporal frequencies. In contrast, at higher temporal frequencies, the L-cone driven responses are larger than the M-cone driven responses. As a result, the L/M amplitude ratios (Fig. 12, upper right plot) are about unity at low temporal frequencies and increase with increasing temporal frequencies. The L-cone driven responses can be 10 and more times larger than the M-cone driven responses in normal trichromats.

Psychophysically measured flicker fusion frequencies for L- and M-cone modulation are about 30 Hz (Huchzermeyer & Kremers, 2016) which is lower than those measured with ERGs although the stimuli for the ERG measurements were full field whereas the psychophysical measurements were performed with an annular test field with a 2° inner and 13° outer diameter. A similar discrepancy was found between RGC responses and psychophysical data, which led to the proposition of the presence of a cortical low-pass filter (Lee et al., 1990b). The cut-off frequency was proposed to be smaller for chromatic than for luminance signals.

Silent substitution stimuli that selectively stimulate the L- or the M-cones are combinations of luminance and chromaticity modulations because the two cone types provide inputs to both luminance and cone opponent retinal pathways. Therefore, ERG responses may reflect the activity in each of these pathways or combinations of the two.

The ERG responses of normal trichromats to L-cone isolating stimuli at high temporal frequencies are generally larger than those to M-cone isolating stimuli with equal cone contrast. However, there is large inter-individual variability. In psychophysical measurements of flicker detection thresholds at high temporal frequencies (> 15 Hz), trichromats are generally more sensitive to L- than to M-cone isolating stimuli but again with considerable inter-individual variability (Huchzermeyer & Kremers, 2016). At these frequencies, the magnocellularly based luminance channel mediates detection (Kremers et al., 1992). Interestingly, the individual L/M-ERG-amplitude ratio is correlated with the individual psychophysical L/M-sensitivity ratio (Kremers et al., 2000). Furthermore, these ratios are correlated with the L/M-ratio of number of cones (Brainard et al., 2000) and of the L/M-ratio of cone pigment densities (Kremers et al., 2000).

These results indicate that the ERGs at high temporal frequencies reflect activity of the luminance pathway. The cone weight in this pathway is determined by the number of cones without additional weighting of the signals. The question is, however, if the correlation between psychophysical and ERG ratios is causal or merely indirect because two independent mechanisms process L- and M-cone inputs in a similar manner.

Additional indications of a causal relationship can be obtained by comparing ERG responses at low and intermediate temporal frequencies (below about 14 Hz) are compared with psychophysical detection sensitivities at low temporal frequencies (typically below about 4 Hz). The ERG amplitude ratio and the sensitivity ratio are about unity for all trichromatic subjects with little inter-individual variability (Kremers et al., 2000; Huchzermeyer & Kremers, 2016). The psychophysical sensitivities are mediated by the parvocellular red-green chromatic channel in which L- and M-cone signals are processed in an antagonistic manner. Thus, the ERG responses most probably reflect activity in this chromatic channel. This notion is further strengthened by the finding that the L- and M-cone driven ERGs have 180° phase differences when the photoreceptors are stimulated in phase, indicating cone opponent post-receptoral processing (see Fig. 12 lower graphs).

In conclusion, the ERG responses to L- and M-cone isolating stimuli indicate that they reflect activity of the luminance pathway at temporal frequencies above about 30 Hz and of the red-green chromatic channel at frequencies below 14 Hz. This agrees with the results obtained with the heterochromatic stimuli as presented in the previous section. Furthermore, the fact that activities of both major retinal pathways are reflected in the ERG indicates that the signals indeed originate in the pathways rather than being merely fortuitous results of analogous processing.

Similar to ERGs to heterochromatic stimuli, silent substitution ERGs have different spatial properties when they reflect different post-receptoral mechanisms: When the responses reflect magnocellular activity (i.e., at high temporal frequencies) then the response amplitudes are positively correlated with stimulus size. In contrast, the parvocellular reflecting responses (i.e., below 14 Hz) do not depend on stimulus size for a large range of stimulus sizes (Jacob et al., 2015). This result is again in agreement with those obtained with heterochromatic stimuli as described in the previous section.

ERG responses to S-cone isolating stimuli can also be recorded. Although S-cone contrasts can often be larger than those for L- or M-cone isolating stimuli (because the L- and M-cone fundamentals strongly overlap while S-cone fundamentals do not show as much overlap, unless melanopsin-containing ipRGCs are also considered) their responses to sine wave stimuli are smaller than those to L- and M-cone isolating stimuli. This is probably caused by their much lower density throughout the retina (Kremers & Pangeni, 2012).

Rod isolating sinusoidal stimuli ERGs using the silent substitution can be measured up to relatively high retinal illuminances (of about 500 photopic td) and without the need of lengthy dark adaptation periods (Maguire et al., 2016). They have a low-pass temporal frequency characteristic at lower retinal illuminances.

Another manner to study rod-driven responses using the silent substitution procedure without the need for lengthy dark adaptation is by using smaller stimulus sizes. If the area surrounding the stimulus is dark then large rod-driven responses can be obtained, probably due to stimulation of the retina surrounding the stimulus through stray light. It was also found that retinitis pigmentosa patients showed strongly decreased response amplitudes to these stimuli (Aher et al., 2018).

### Responses to sawtooth and square wave stimuli

Stimuli with sawtooth temporal profiles may be used to separate On- and Off-center RGC responses. The On-pathway is mainly stimulated by rapid-on ramp-off stimuli whereas rapid-off ramp-on stimuli favor responses in the Off-pathway. The bias is caused by the fact that the change in firing frequency is larger for excitatory than for inhibitory stimuli (the latter is limited by the spontaneous activity of the cells). In addition, the rapid on−/ offsets in the stimuli elicit larger responses than the ramps. Such a bias may be less strong or even absent for bipolar cells that transmit information through graded de- and hyper-polarizations. Since bipolar cell activity may have a large influence on the ERGs (Frishman, 2006), the On–Off asymmetry may be smaller in the ERG. The d-wave in the Off-responses is to a large extent mirror images of the a-wave in the On-responses (Pangeni et al., 2012; Tsai et al., 2016) indicating that these components originate early in the visual cascade where processing is still quite linear. This is in agreement with the notion that the two components mainly originate in the photoreceptors (Frishman, 2006). Nevertheless, ERG responses to rapid-on and rapid-off sawtooth show different characteristics and are not mirror images of each other, as would be expected for a linear system. This asymmetry is probably also the origin of the pattern ERG (Bach & Hoffmann, 2006).

Square waves or long flashes can be also be used to study On- and Off- responses (Sustar et al., 2018). However, the On- and the Off-phases must be long enough to reflect the complete response, so that rapid adaptation processes may interfere. On–Off-asymmetries are probably also the origin of the photopic hill effect where the On- and Off-responses are thought to have different effects on the b-wave amplitudes of flash ERGs. The result is a decrease in b-wave amplitude with increasing flash strength when strong flashes are employed (Ueno et al., 2004; Hamilton et al., 2007). Finally, On–Off phase differences are thought to cause the strong minimum at 12 Hz in the fundamental component of the response to luminance sinusoidal stimuli as described in section “Luminance stimuli at different temporal frequencies” (Kondo & Sieving, 2001).

It was found that On- and Off-responses were affected by different disease such as complete or incomplete CSNB with Schubert-Bornschein type ERGs (Miyake et al., 1987), Duchenne Muscular Dystrophy (Barboni et al., 2013) and X-linked Retinoschisis (Zobor et al., 2023). In these disorders, the signal transmission at the synapse between photoreceptors and bipolar cells is affected. Asymmetric On–Off response changes were also found for glaucoma (Horn et al., 2011; Pangeni et al., 2012). The origin of these changes is not fully understood.

**Figure 13.**
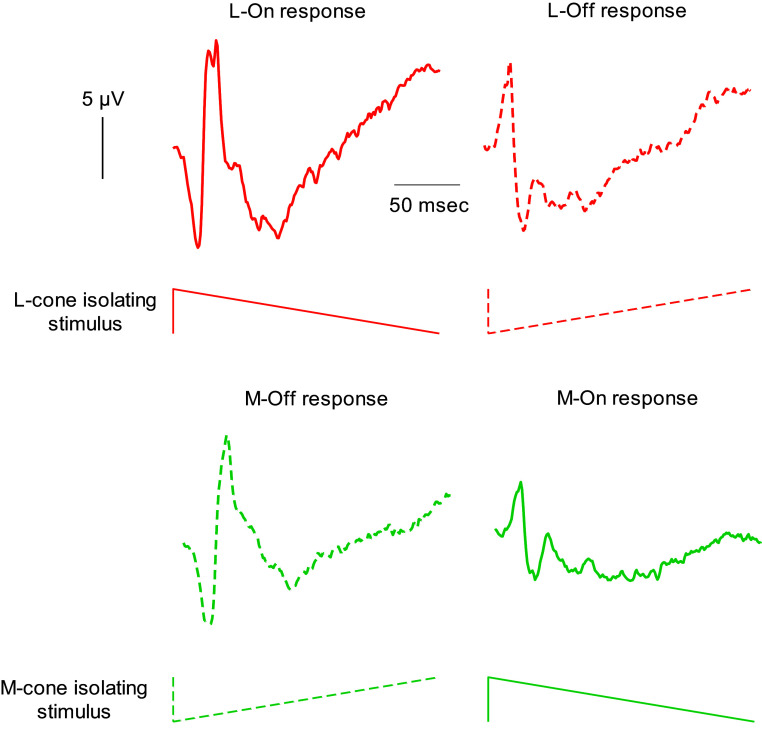
ERG responses to L- and M-cone isolating sawtooth stimuli. The upper curves show original responses to L-cone isolating stimuli in a trichromatic subject (Left: response to rapid increases of L-cone excitation; right: response to rapid decreases of L-cone excitation). The lower curves show the responses to M-cone isolating stimuli (Left: response to rapid M-cone excitation decreases; right: response to rapid M-cone excitation increases). Please observe that the L-On and the M-Off responses resemble each other. Similarly, the L-Off and the M-On responses show strong similarities. Adapted from Kremers et al. (2014).

Sawtooth and square wave stimuli can be combined with the silent substitution method. Similar to sine waves, contrast, temporal frequency, and photoreceptor contributions can be varied without changing the mean state of adaptation. Again, this is not the case for flashes. Responses to low-frequency square waves may also be influenced by fast adaptation. L- and M-cone driven responses to square wave (McKeefry et al., 2014) and sawtooth stimuli (Kremers et al., 2014; Tsai et al., 2016) reflect cone opponent processing because, the L-cone On-response resembles the M-cone Off-response and vice versa (see Fig. 13).

S-cone driven responses to square wave stimuli are small (as they are to sine wave stimuli). They show a clear On-response (i.e., a response to an increase in S-cone excitation), but the Off-response is absent in normal subjects. A validation of the stimulus was provided by the absence of response in S-cone monochromats. On the other hand, a patient with enhanced S-cone syndrome showed greatly altered responses (Maguire et al., 2018).

Rod isolating square wave responses show an On-response and a mirror imaged Off-response as well as a slow negativity after rod excitation increase. Rod monochromats showed response waveforms that were similar to those of trichromats, validating that the stimuli isolated the responses of the rods. Rod bipolar cells are only of the On-type. The symmetry between On- and Off-responses therefore suggests that push-pull mechanisms are involved. This notion is strengthened by ERGs recordings in patients with CSNB, where the transmission to rod bipolar cells is disturbed. In these patients On- and Off-responses are absent and only the slow negativity is observed (Maguire et al., 2017).

It can be concluded that photoreceptor-isolating sawtooth and square wave responses display characteristic changes in different patient groups, indicating that they can be used for diagnosing and monitoring these diseases.

### White noise stimuli

White noise stimulation has been frequently used to characterize responding mechanisms in physiological experiments (Marmarelis & Marmarelis, 1978), including the visual system (see e.g., Field et al., 2010). To our knowledge, it has been introduced only fairly recently in ERG recordings where the stimuli, similar as with other repetitive stimuli, are presented repeatedly in sweep periods (Saul & Still, 2016; Zele et al., 2017; Adhikari et al., 2019; Kremers et al., 2022). White noise stimuli contain all frequencies with equal amplitudes and randomized phases. The frequency range can be limited to those frequencies to which the system responds. This generally increases the SNRs of the recordings. Furthermore, the amplitude spectrum can be adapted so that it more closely resembles the spectra of natural scenes.

The use of white noise stimuli can be an efficient way to characterize the mechanisms generating the ERG. The cross-correlation between stimulus and response results in the impulse response function (IRF), which is the response of the system to a short flash assuming that the system is purely linear. The differences between the IRFs and the recorded flash ERGs provide indications about the nonlinearities that are involved in the flash ERGs. These differences are substantial. For instance, the oscillatory potentials (OPs), that can be large in the flash ERGs, are absent in the IRF (Zele et al., 2017; Kremers et al., 2022). This indicates that the OPs reflect strongly nonlinear processes in the retina. As mentioned above (see section “The flash ERG and psychophysical data”), the OPs may indeed be related to the bursty responses of RGCs. The temporal white noise stimulus is generally more convenient for the observer because of the lower contrasts.

White noise stimuli can also be used to derive the modulation transfer function (MTF) of the system’s linear response through the Fourier transform of the IRF. The MTF describes the responses to sinusoidal stimuli of different temporal frequencies. Again, a comparison with real measurements (as e.g., described in section “ERG responses to sinusoidal stimuli”) can give indications about present nonlinearities that are involved. Thus, white noise stimuli in ERG recordings can be very useful to efficiently characterize several aspects of the responding mechanisms in the retina.

**Figure 14.**
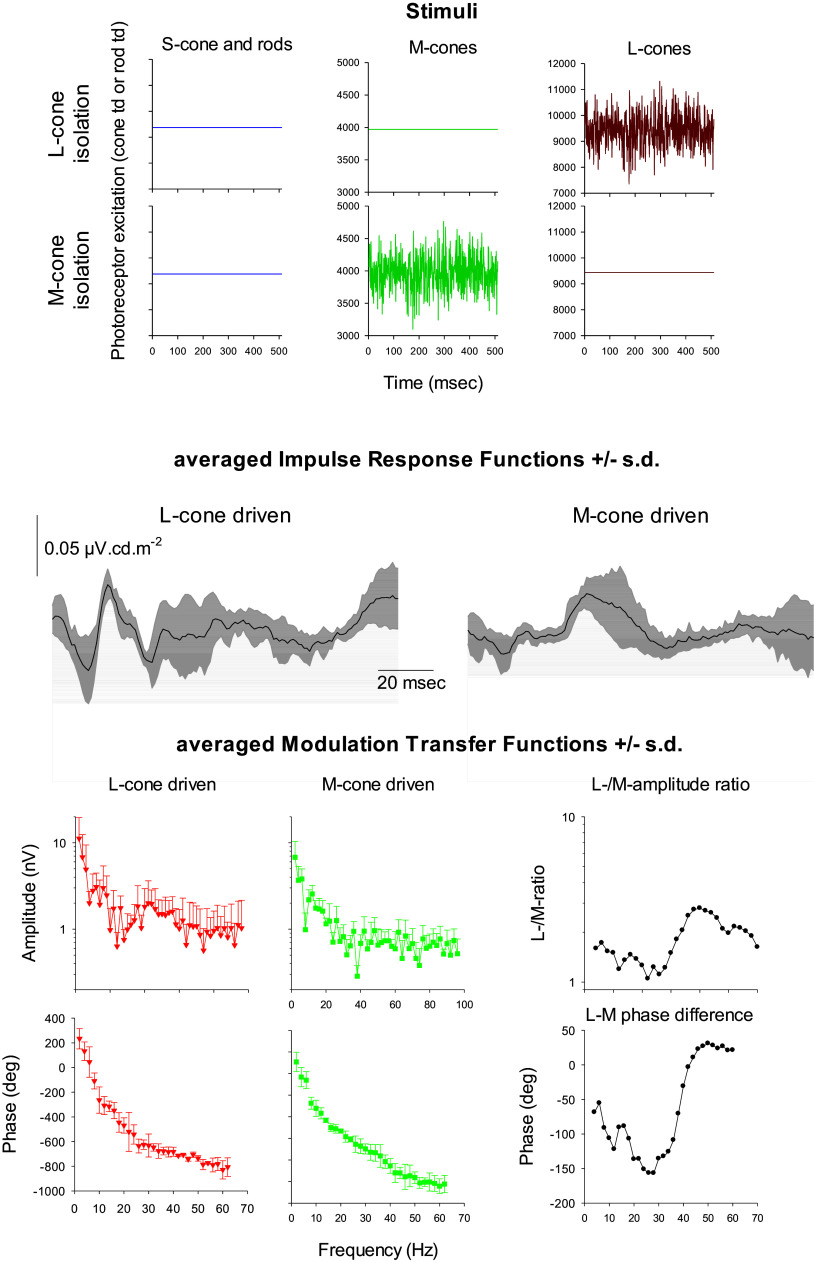
Results of ERG measurements in macaque monkeys using L- and M-cone isolating white noise stimuli. The upper two rows show the excitation of the M-cones as a function of time within a 512 msec cycle. These cycles were presented repetitively. The excitations of only the L-cones (upper row) or only the M-cones (second row) were modulated according to a white noise profile. The excitations in the other photoreceptors were constant (i.e., their output was not modulated). Third row: the cross-correlation between stimulus and ERG response resulted in the L- (left) and M-cone (right) driven impulse response functions (IRF). Observe the fundamental differences between the two. Fourth and fifth rows: The Fourier transform of the IRFs resulted in the modulation transfer functions (MTFs) with separate plots for amplitude (fourth row) and phase (fifth row) for L- (left) and M-cone (middle) driven responses. The L−/M-amplitude ratio (fourth-row right plot) and the L-M phase differences (fifth-row right plot) were derived.

Temporal white noise stimuli can be combined with the silent substitution method to isolate the responses originating in different photoreceptor types. A sketch of L- and M-cone isolating white noise stimuli is shown in Fig. 14 (upper two rows). The L- and M-cone driven white noise IRFs (Fig. 14 middle row) and MTFs (Fig. 14 lower two rows) were derived from the ERGs measured in monkeys (Kremers et al., 2022). As with sinusoidal stimuli (Kremers et al., 2021a), luminance and chromatic reflecting ERG components could be identified: The L−/M-amplitude ratio is about unity at frequencies below about 25 Hz. At these frequencies the absolute value of the phase difference between L- and M-cone driven responses are large (the values are negative indicating that the M-cone driven response lead the L-cone driven responses). These are properties of the L−/M-cone opponent pathway. For higher frequencies, the L-M-ratio increases and the absolute phase difference decreases, indicating an increasing role of the luminance pathway (cf. with the sine wave data presented in Fig. 12). In conclusion, white noise stimuli can be an efficient way to characterize the properties ERG generating mechanisms because many properties can be characterized that would need substantially more time with other procedures.

## Future perspectives

In the present article, we propose that ERG responses to repetitive stimuli can lead to a substantial extension of the current knowledge on visual signal processing in the retina that can be used both in basic and clinical research evidence. We provided evidence that supports this proposition from different sources. We are aware that many aspects and implications of the current knowledge and data are not fully explored yet, particularly for clinical research. In this section, we identify some issues that may be of interest for future research, without claiming to be complete or even exhaustive.

### Interest for basic research

#### Development of color vision

Periodic ERGs could contribute to a better understanding of how color vision develops in life. We showed that the ERGs may capture the activity of L-M cone opponency in the red-green chromatic channel. Cone opponency most probably depends on the spectral differences between cone fundamentals. It would be interesting to explore the relationship between the genotype and phenotype of cone pigment absorption spectra on the one hand and the cone-driven opponent signals as measured with the ERGs on the other hand. From the L- and M-cone pigment genes, the effects on the cone fundamentals and thus on their spectral differences can be inferred. By selecting anomalous trichromats with variable spectral differences and measuring the cone opponent signals in the ERGs, a direct relationship can be obtained. One possibility is that there is a continuous change in cone opponent signals with the spectral differences. Another possibility is that a minimal spectral difference is required for cone opponency to fully develop.

#### ERG signals of S versus L/M opponency

So far, mainly L−/M-cone (red-green) opponent signals have been explored and measured. It would be interesting if signals of S- versus LM-opponent (koniocellular; putatively blue-yellow) pathways can be retraced in ERG signals. S-cone signals have been explored (Maguire et al., 2018) but the signals are relatively small probably due to the sparsity of S-cones. As a result, post-receptoral processing of S- versus LM-opponency may be similarly difficult to detect in the ERG.

#### ERGs driven by internally photosensitive retinal ganglion cells

Intrinsically photosensitive retinal ganglion cells (IpRGCs) have recently raised strong interest because they are believed to also have an influence on visual perception (see e.g., Zele et al., 2019). It has been reported that white noise stimuli that isolate the responses of ipRGCs can elicit ERGs (Adhikari et al., 2019). However, these recordings should be more thoroughly validated. Perfect isolation of ipRGCs may be difficult to obtain because their spectral sensitivities strongly overlap with those of rods and S-cones. Small deviations from perfect isolation (e.g., due to eccentricity-dependent changes in the fundamentals) may introduce residual responses in the rods and/or the S-cones that may easily outweigh the ipRGC-driven responses because they are more abundantly present (particularly the rods) and because, in contrast to ipRGCs, the rods and S-cones elicit responses in bipolar cells, which are known to contribute substantially to the generation of the ERG (Frishman, 2006). If it can be unequivocally established that ipRGC-driven ERGs can be reliably measured, then the results can be compared with pupillometric and psychophysical data and information about retinal processing can be obtained.

#### The influence of adaptation

The influence of adaptation processes can be studied with repetitive stimuli. With flashed stimuli the mean luminance and/or the mean chromaticity change with flash strength and with flash frequency. It is therefore not possible to study the influence of state of adaptation with flash ERGs. With repetitive stimuli in which luminance and chromaticity are symmetrically distributed around the mean (which is the case for all repetitive stimuli that were described above), the state of adaptation can be studied without confounding with other stimulus attributes when the changes are too fast relative to the time constant for adaptation. We have studied cone selective adaptation on the ERG before using a CRT screen as stimulator (Kremers et al., 2003). More detailed studies on the influence of photoreceptor selective adaptation on the ERG responses driven by the same or by another photoreceptor may give information on the processing of photoreceptor signals in the retina.

#### Interactions between pathways

Interactions between pathways can be studied by comparing the responses to stimuli that selectively stimulate single pathways with those in which the ERGs are determined by simultaneous activities of different pathways. We found before that ERGs that simultaneously reflect cone opponent and luminance activity can be satisfactorily described by a linear vector addition of the separate activities (Kremers et al., 2021b). This could be extended, for instance, by adapting one pathway and measuring its influence on another pathway. If more pathways can be measured (e.g., S versus L/M opponency, rod-driven, ipRGC-driven), their mutual interactions could be studied.

#### Spatial properties of the ERGs reflecting different post-receptoral pathways

As described before, the cone opponency and luminance reflecting ERGs have very different dependencies on stimulus size and its retinal position (Jacob et al., 2015; Martins et al., 2016; Vidal et al., 2021; Kremers et al., 2021b). These spatial properties do not seem to have a connection with the receptive field properties of the neurons belonging to the different pathways but can be used to characterize the different ERG signals. It remains to be established if the number of stimulated neurons plays a role in spatial integration of ERG-generating mechanisms.

### Interest for clinical research

The described developments in basic research and their correlations with psychophysical data not only provide new insights into basic processes in the visual system but also open up exciting possibilities for further exploration in clinical research.

The progress in gene- and cell-based therapies for inherited retinal diseases has renewed interest in measuring responses of different photoreceptor types (Cideciyan et al., 2021). Methods based on retinal adaptation are still the most frequently used (Cideciyan et al., 2021). However, the silent substitution strategy, with its unique ability to control the modulation of different photoreceptor subtypes *under identical retinal adaptation*, offers a promising solution to the known limitations of these techniques (Simunovic et al., 2016, 2021).

Histological (Agorastos & Huber, 2011) and pupillometric (Feigl et al., 2011; Kankipati et al., 2011) studies have demonstrated that glaucoma affects intrinsic photosensitive retinal ganglion cells (ipRGCs). Silent-substitution-ERGs may clarify the clinical relevance of these findings. Studying how different RGC types are affected in glaucoma supports developing functional endpoints in trials on neuroprotective therapies in glaucoma (Kim et al., 2021) and enable addressing disturbances of the circadian rhythm in glaucoma patients (Agorastos & Huber, 2011).

As shown above, white noise stimuli are generally more convenient for the patient than flases may. The ERGs elicited by white noise stimuli may help to elucidate retinal processing, that are affected in diseases like diabetic retinopathy in which glial cells are involved (Coughlin et al., 2017). There is a great interest in studying how metabolic control and neuroprotective strategies help retain retinal function in early diabetes (Simo & Hernandez, 2022), especially because visual acuity is altered late in the course of the disease and earlier changes may already considerably affect quality of life (Glassman et al., 2024).

Lastly, the use of periodic stimuli may also facilitate recording ERGs in clinical practice because patients tolerate periodic stimuli better than bright flashes that can be quite unpleasant. In addition, Fourier or white noise analysis of periodic stimuli can lead to outcomes that are more robust and less prone to artifacts. Therefore, using periodic stimuli may also improve the quality of ERGs recorded with skin electrodes, which are better tolerated than, for example, contact lens electrodes.

The comparisons between ERG and psychophysical data in different patient groups with those obtained in normal control subjects may give useful information about which pathways may be affected by the disease and may also point at the involved pathological mechanisms. The data may also be used for developing better and more robust tools for diagnosing and monitoring the diseases. It may be very helpful to select and standardize procedures that can be introduced in clinical routine and enabling comparisons of results obtained in different institutions. The temporal and spectral requirements of the equipment are probably stricter for quantifying stimulus strengths in physiological terms (photoreceptor td for mean excitation and photoreceptor contrast for excitation modulation) but these issues can be handled relatively easily nowadays.

The silent substitution method will be an interesting extension for clinical studies. The isolation of the responses of single photoreceptor types or of well-defined response combinations will enable a better insight in pathophysiological processes. Different photoreceptor responses can be isolated without changing the state of adaptation so that the outcomes of the different conditions can be compared with each other. In addition, the state of adaptation can be an additional invariant that can be studied. The combination with periodic stimuli enables the possibility of frequency tagging and Fourier analysis (Norcia et al., 2015) thereby ensuring acceptable SNRs of the recordings. Tutorials and tools for the calculation of the silent substitution conditions are available (Martin et al., 2023; Nugent et al., 2023). Currently, the International Society for Clinical Electrophysiology of Vision (ISCEV) is establishing an extended protocol for the use of the silent substitution method for clinical application.

## Concluding remarks

It was the intention of the present review to show that repetitive stimulation for ERG recordings can be an important extension of the current conventional flash ERGs, which helps (1) to characterize ERG generating mechanisms, (2) to correlate ERGs with neurophysiological activity in retino-geniculate pathways and with psychophysical data on retinal visual processing, and (3) to increase the clinical relevance of ERG recordings. We are convinced that developments in non-invasive electrophysiological research described in this review can have a significant impact on better understanding of the function and dysfunction of the retina.
